# Mechanism of the Microstructural Evolution of 18Cr2Ni4WA Steel during Vacuum Low-Pressure Carburizing Heat Treatment and Its Effect on Case Hardness

**DOI:** 10.3390/ma13102352

**Published:** 2020-05-20

**Authors:** Bin Wang, Yanping He, Ye Liu, Yong Tian, Jinglin You, Zhaodong Wang, Guodong Wang

**Affiliations:** 1 State Key Laboratory of Rolling and Automation, Northeastern University, Shenyang 110819, China; 15734069747@139.com (Y.H.); ly987109166@163.com (Y.L.); zhdwang@mail.neu.edu.cn (Z.W.); wanggd@mail.neu.edu.cn (G.W.); 2 State Key Laboratory of Advanced Special Steel, Shanghai Key Laboratory of Advanced Ferrometallurgy, School of Materials Science and Engineering, Shanghai University, Shanghai 200072, China; jlyou@staff.shu.edu.cn

**Keywords:** vacuum low-pressure carburizing, 18Cr2Ni4WA steel, low-temperature tempering, retained austenite, case hardness

## Abstract

In this study, vacuum low-pressure carburizing heat treatments were carried out on 18Cr2Ni4WA case-carburized alloy steel. The evolution and phase transformation mechanism of the microstructure of the carburized layer during low-temperature tempering and its effect on the surface hardness were studied. The results showed that the carburized layer of the 18Cr2Ni4WA steel was composed of a large quantity of martensite and retained austenite. The type of martensite matrix changed from acicular martensite to lath martensite from the surface to the core. The hardness of the carburized layer gradually decreased as the carbon content decreased. A thermodynamic model was used to show that the low-carbon retained austenite was easier to transform into martensite at lower temperatures, since the high-carbon retained austenite was more thermally stable than the low-carbon retained austenite. The mechanical stability—not the thermal stability—of the retained austenite in the carburized layer dominated after carburizing and quenching, and cryogenic treatment had a limited effect on promoting the martensite formation. During low-temperature tempering, the solid-solution carbon content of the martensite decreased, the compressive stress on the retained austenite was reduced and the mechanical stability of the retained austenite decreased. Therefore, during cooling after low-temperature tempering, the low-carbon retained austenite transformed into martensite, whereas the high-carbon retained austenite still remained in the microstructure. The changes in the martensite matrix hardness had a far greater effect than the transformation of the retained austenite to martensite on the case hardness of the carburized layer.

## 1. Introduction

The 18Cr2Ni4WA steel is a low-carbon alloy steel with a good hardenability. After carburizing, a high-strength surface and high-toughness core can be achieved simultaneously. This steel is widely used under high-speed and heavy-duty conditions for the main load-bearing parts of aviation engines, gas turbines and other large machines, such as crankshafts, gear teeth, adapter plates and flanges [1,2,3]. Carburizing this material results in a high surface carbon content, a martensite matrix with a high strength and hardness and a large quantity of retained austenite, which can be obtained during quenching or even under air cooling conditions [4]. Low-temperature tempering is subsequently used to reduce or eliminate internal stresses in the quenched microstructure to improve the dimensional accuracy and strength of the workpieces. Over the 150 °C–250 °C range generally used for low-temperature tempering, the migration of carbon atoms in the carburized layer over short distances results in a series of microstructural changes. The microstructural evolution that occurs during carburizing heat treatments ultimately determines the hardness and gradient distribution of the carburized layer, which directly affects the service performance under surface wear and fatigue [5,6,7,8].

Tempering is generally considered to reduce the stability of the retained austenite, which promotes the transformation to martensite during subsequent cooling to increase the surface hardness [9,10]. Shi [11] proposed that the retained austenite in 18Cr2Ni4W steel could transform into secondary martensite during tempering and cooling. Chen et al. [12] studied the tempering of GCr15 bearing steel and found that the thermal stability of austenite depended on the combined effects of the carbon distribution in austenite and carbide precipitation. Nam et al. [13] found that alloying elements did not affect the decomposition of the retained austenite in induction hardened-and-tempered steels. Wu et al. [14] conducted a detailed study on microstructural evolution and the effects of alloying elements during the tempering of martensite. Feng et al. [15] proposed that during low- and medium-temperature tempering, carbon discharge from martensite to nearby austenite increased the carbon content and thermal stability of the retained austenite. Chen et al. [16] found that the low-temperature cryogenic treatment of 40CrNiMoA steel promoted the transformation of retained austenite to martensite and the precipitation and uniform distribution of carbides. The martensite substructure was also refined, and the steel hardness was improved. Therefore, an in-depth study of the mechanism of the microstructural phase transformation that occurs during a carburizing heat treatment can provide important guidance to control carburization and optimize the material performance.

In this study, vacuum low-pressure carburization was carried out on 18Cr2Ni4WA steel. Multistage pulse carburization was used to precisely control the depth of the carburized layer and the carbon content and prevent intergranular oxidation, thereby forming a uniform and stable carbon gradient distribution. A thermodynamic model was used to calculate the driving force for the austenite phase transformation and decomposition. These results were used to study the migration and diffusion behavior of carbon in the martensite in the carburized layer microstructure of 18Cr2Ni4WA steel, the conditions for the transformation of retained austenite and the subsequent effects on the hardness of the carburized layer. This study can provide a theoretical reference for investigating the microstructural evolution of case-carburized steels during carburizing and tempering heat treatments.

## 2. Experimental Materials and Methods of Vacuum Low-Pressure Carburization

Th experiments were performed on 18Cr2Ni4WA steel, a heavy-duty gear steel (Fushun Special Steel, Fushun, China). The steel was processed into a φ22 mm bar sample after vacuum induction melting, cold drawing and annealing. The main chemical components of 18Cr2Ni4WA steel are shown in Table 1.

**Figure 1:**
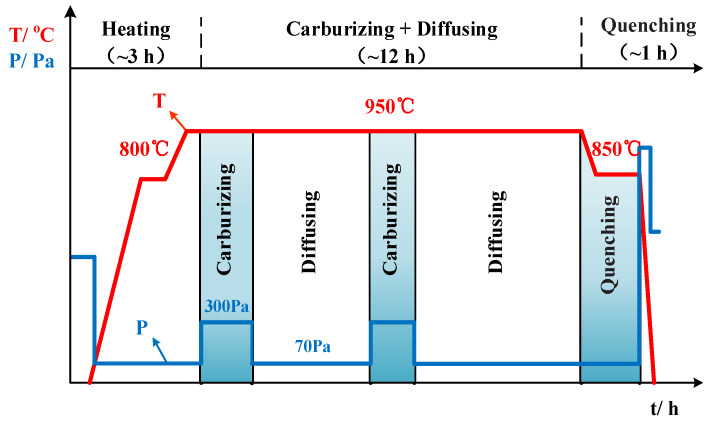
Schematic diagram of the vacuum low-pressure carburizing process.

A schematic of the vacuum low-pressure carburizing process is shown in Figure 1. The sample was placed in a DBVC-433 vacuum carburizing device developed by Northeastern University (Shenyang, China) for two-stage heating at a rate of 10 °C/min. The temperature was held at 800 °C for a period of time, after which the austenitizing temperature was held at 950 °C for 1 h; then, the sample was carburized. The experiment was carried out using the “carburizing + diffusing” multistage pulse method. The carburizing medium was C_2_H_2_ gas introduced with a flow rate of 10 L/min and a carburizing pressure of 300 Pa. To prevent the cementite from affecting the evolution and hardness of the matrix microstructure, the carbon content on the surface of the carburized layer was set to 0.7%. During the diffusing pulse, the gas in the furnace was pumped out and the furnace was filled with nitrogen at a pressure of 70 Pa. The “carburizing + diffusing” cycle was repeated eight times. The total carburizing time was 12 h for a carburizing depth of 1.3 mm.

**Figure 2:**
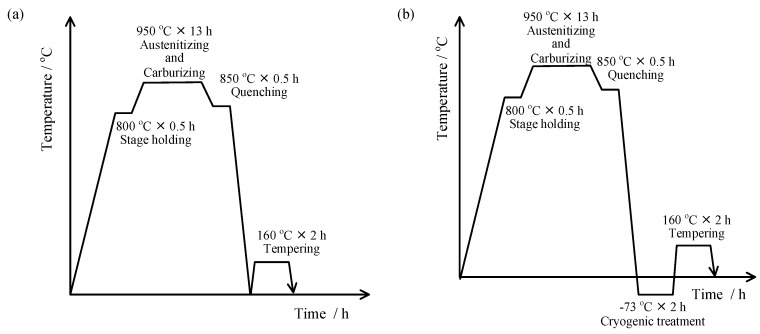
Schematic diagram of the carburizing heat treatment process of the 18Cr2Ni4WA steel: (**a**) process 1: quenching + tempering; (**b**) process 2: quenching + cryogenic treatment + tempering.

The post-carburizing heat treatment is shown in Figure 2. After carburizing, the furnace was cooled to 850 °C, and the temperature was held for 0.5 h. Vacuum oil quenching was performed at an oil quenching rate of approximately 75 °C/s. The temperature of the oil was 25 °C and the quenching time was approximately 15 min. Subsequently, low-temperature tempering was conducted at 160 °C (process 1) for 2 h. A cryogenic treatment (process 2) was performed as a supplemental experiment prior to the low-temperature tempering: the temperature was maintained at −73 °C for 2 h using a mixed dry ice/ethanol solution as the cooling medium.

After the entire treatment was completed, the microstructure and properties of the carburized steel were analyzed. Following erosion by 4% nitric acid in ethanol, the microstructure of the carburized layer was observed using a BX53M optical microscope (Olympus, Tokyo, Japan) and an Ultra 55 scanning electron microscope (SEM, Zeiss, Germany). The microhardness of the material was measured every 0.1 mm along the direction of the carburized layer by using an FM-700 Vickers hardness tester (Future-Tech, Kawasaki, Japan).

The microstructure was observed via transmission electron microscopy (TEM) using a TECNAI-G^2^ 20F (FEI, Hillsboro, OR, USA). To prepare the TEM sample, mechanical polishing was used to reduce the sample thickness to 50 μm, which was followed by twin-jet polishing. The electrolyte solution was 10% (by volume) perchloric acid in ethanol, the polishing temperature was −25 °C, the polishing voltage was 38 V and the current was 55 mA.

The X-ray diffraction (XRD) sample size was 10 mm × 10 mm × 5 mm. Twin-jet polishing was followed by electrolytic polishing using the following parameters: a voltage of 20 V, a current of 1.1–1.2 A and an electrolytic polishing time of 20 s. The XRD was performed using a D8 Advance X-ray diffractometer (Bruker, Leipzig, Germany) with a diffraction angle of 40–100° and a speed of 2°/min.

A standard round bar specimen with a length of 100 mm was also carburized to determine the carbon content distribution of the carburized layer. Iron chips were obtained from the carburized specimen by cutting layer by layer with a 0.1 mm layer depth. Then, the carbon content of the iron chips obtained from each layer was measured using a CS230 carbon–sulfur analyzer (LECO, St Joseph, MI, USA).

## 3. Results: Microstructure and Performance after Carburizing and Quenching

**Figure 3:**
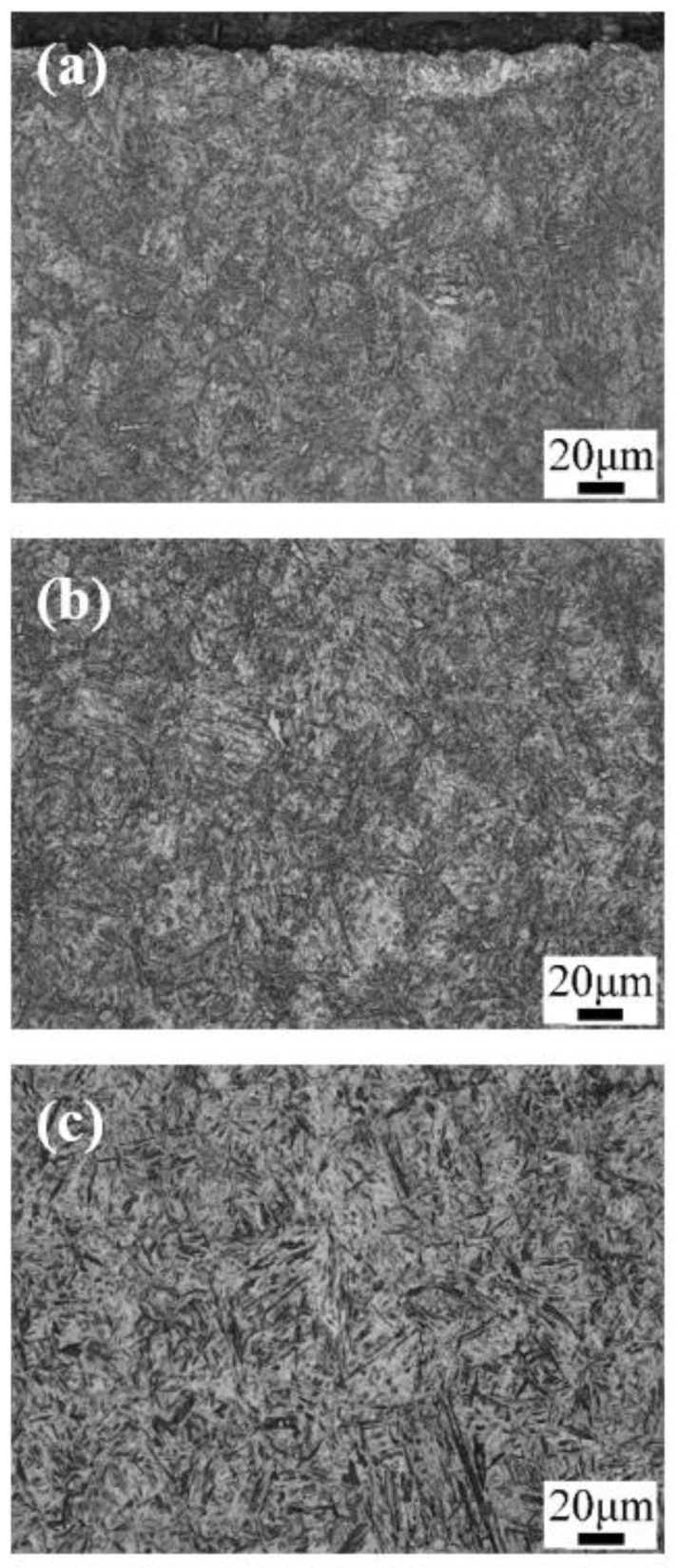
Optical micrographs of the microstructure after carburizing and quenching: (**a**) the surface; (**b**) the subsurface; and (**c**) the core.

**Figure 4:**
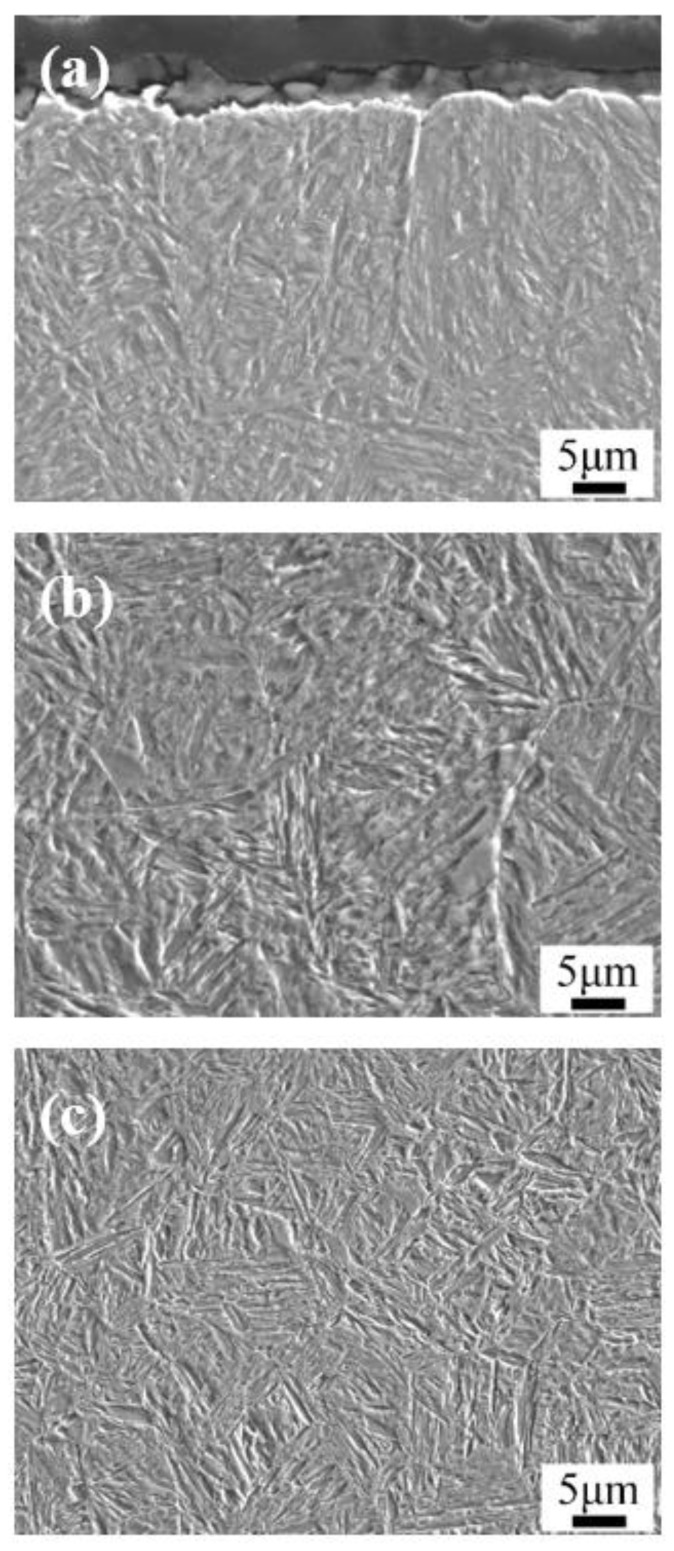
SEM images of the microstructures after carburizing and quenching: (**a**) the surface; (**b**) the subsurface; and (**c**) the core.

Figure 3 and Figure 4 show the optical micrographs and SEM images of the microstructure after carburizing and quenching, respectively. The surface microstructure of the carburized layer was composed of small acicular martensite particles, and no apparent carbide precipitation was observed. The subsurface layer was composed of a large quantity of acicular martensite and a small quantity of lath martensite. As the subsurface had a lower carbon content than the surface layer, there were large, relatively coarse needle-like martensite structures. Further reduction in the carbon content in the core microstructure resulted in the formation of lath martensite. The carburized layer had a martensite matrix with a fine, uniform microstructure and high hardness.

**Figure 5:**
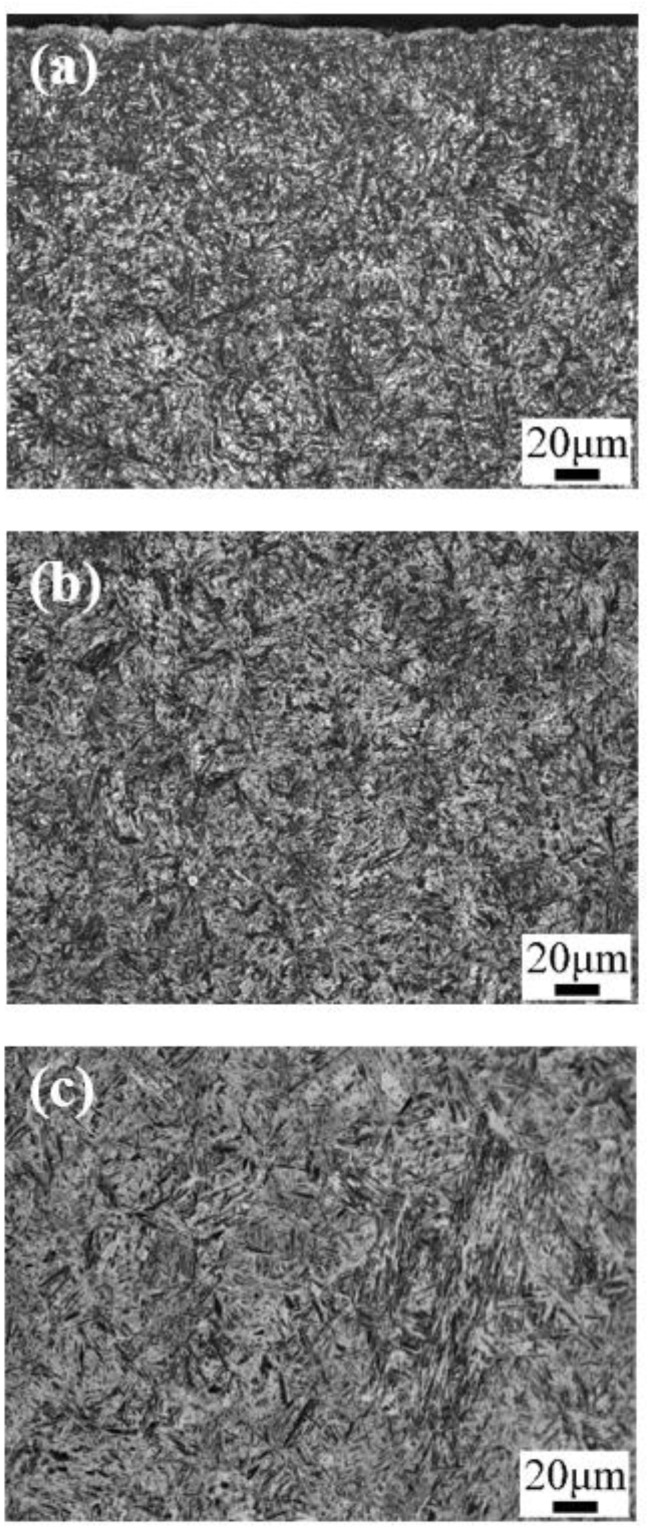
Optical micrographs of the microstructures after low-temperature tempering: (**a**) the surface; (**b**) the subsurface; and (**c**) the core.

**Figure 6:**
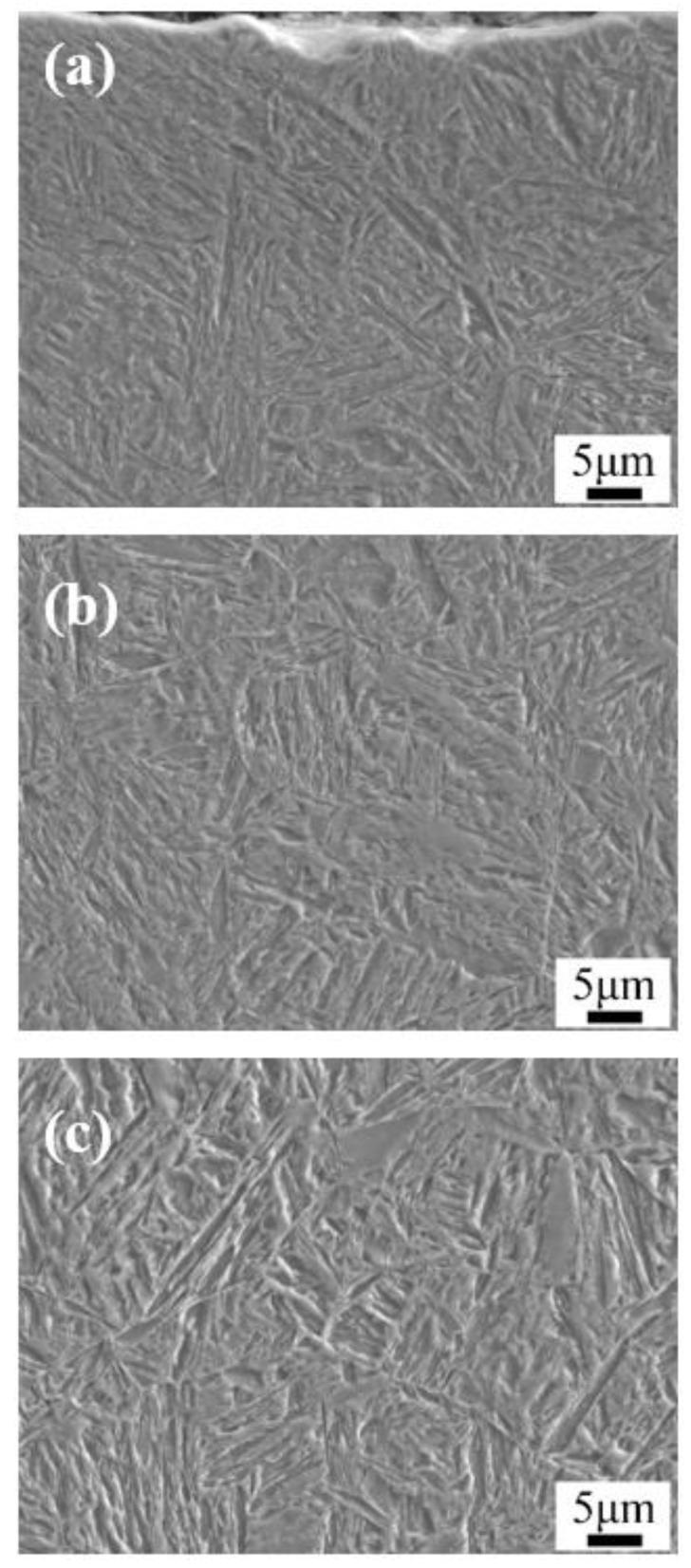
SEM images of the microstructures after low-temperature tempering: (**a**) the surface; (**b**) the subsurface; and (**c**) the core.

Figure 5 and Figure 6 are the optical micrographs and SEM images of the microstructure after low-temperature tempering, respectively. The matrix microstructure gradually transformed from high-carbon acicular martensite in the surface layer to low-carbon lath martensite in the core. No distinct carbide precipitation or change in the microstructure type was observed after quenching, and the substructure appeared slightly large.

**Figure 7:**
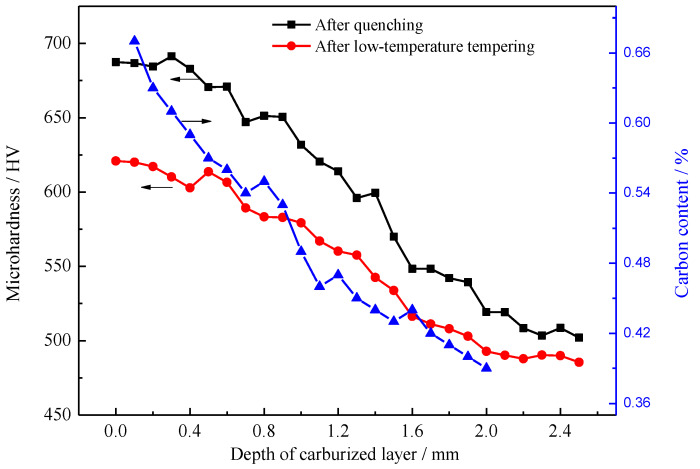
Carbon content distribution and the hardness gradient of the carburized layer.

The carbon content distribution and the hardness gradient of the carburized layer of the 18Cr2Ni4WA steel after quenching are shown in Figure 7. According to the Chinese national standard GB/T 9450-2005 [17], the hardened layer produced by carburizing and quenching was defined as the vertical distance from the surface to a depth with a Vickers hardness of 550 HV. Therefore, the depth of the carburized layer after carburizing was 1.35 mm, which corresponds to 96% of the pre-set carburized layer depth. After quenching, the surface of the carburized layer had a 0.67% carbon content. The measurements of the carburized layer were consistent with the pre-set values. Vacuum low-pressure carburization resulted in a good carbon content distribution in the carburized layer. The carbon content of the carburized layer decreased gradually and evenly, resulting in a uniform hardness gradient of the carburized layer.

The hardness gradient curve shows that the carbon content gradually decreased with increasing depths in the carburized layer and the high-carbon acicular martensite in the surface layer was transformed into low-carbon lath martensite in the core. The overall hardness of the layer was gradually reduced, with small fluctuations in the hardness. After carburizing and quenching, the surface hardness of the carburized layer of the sample was 687 HV and the hardness of the core at a 2.0 mm depth was 519 HV. The maximum surface hardness of the carburized and quenched + low-temperature-tempered samples was 620 HV, and the hardness of the core at a 2.0 mm depth was 485 HV. The hardness gradient shows that the hardness of the carburized layer was relatively high after quenching. However, the surface hardness of the carburized layer was reduced by 67 HV and the hardness of the core was reduced by 34 HV after low-temperature tempering. After low-temperature tempering, the overall hardness of the carburized layer decreased considerably. To explain this result, the transformation process of martensite and the retained austenite in the carburized layer and the diffusion behavior of carbon atoms during low-temperature tempering are analyzed in depth below.

## 4. Discussion

### 4.1. Effect of Low-Temperature Tempering on the Microstructure and Case Hardness

**Figure 8:**
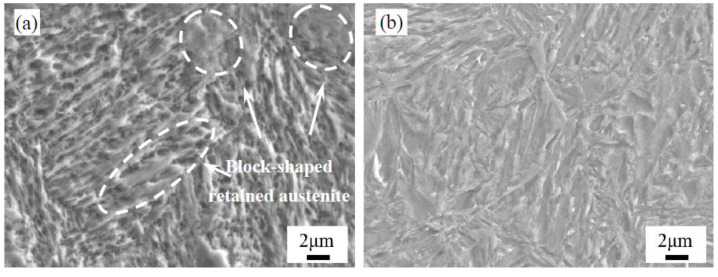
SEM images of the morphology of the carburized layer at a 0.3 mm depth for process 1: (**a**) after quenching; and (**b**) after the low-temperature tempering.

Figure 8 shows the SEM images of the morphology of the carburized layer at a 0.3 mm depth after quenching and low-temperature tempering. The quenched microstructure was mainly composed of acicular and lath martensite + retained austenite. There was a quantity of block-shaped retained austenite between the martensite matrices. After low-temperature tempering, the main microstructure remained as martensite + retained austenite. Thus, compared to the carburizing and quenching, the low-temperature tempering resulted in the disappearance of block-shaped retained austenite. A few fine carbides were present between the martensite needles and the laths after low-temperature tempering.

**Figure 9:**
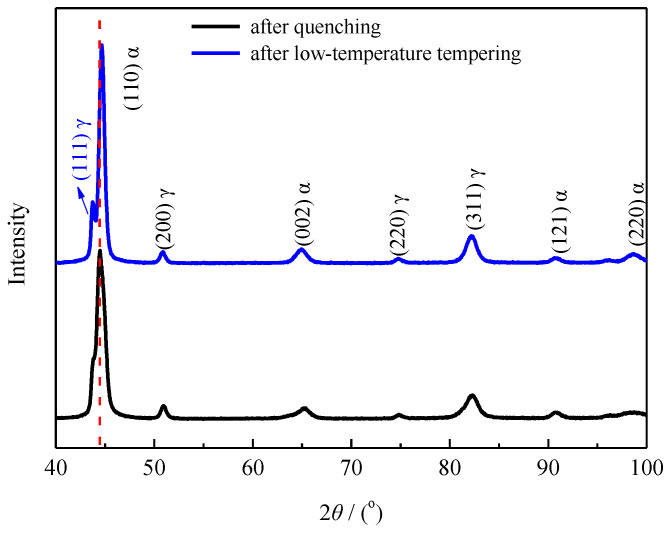
XRD pattern of the carburized layer at a 0.3 mm depth for process 1.

Figure 9 shows the XRD results obtained for the microstructure of the carburized layer at a 0.3 mm depth after carburizing/quenching and quenching + low-temperature tempering.

Using the standard Powder Diffraction File card as a reference, the matrices of the carburized layer of the sample at a 0.3 mm depth before and after low-temperature tempering were determined to contain both martensite and austenite. The strongest XRD peak was assigned to the martensite (110) crystal plane and no carbide peaks were observed. The low carbide content and volume prevented the detection of carbide XRD peaks. The results were consistent with the SEM morphological results. Compared with the XRD peak obtained for the quenched microstructure, the XRD peak of the martensite (110) crystal plane after low-temperature tempering shifted to higher angles and was well separated from the XRD peak of the austenite (111) crystal plane, which therefore appeared distinct.

The XRD data were processed using Equation (1) to calculate the volume fraction of retained austenite [18]:

(1) $$V_{\gamma }^{}=\frac{1.4I_{\gamma }^{}}{I_{\alpha }^{}+1.4I_{\gamma }^{}}$$

where $V_{\gamma }^{}$ is the volume fraction of the retained austenite; $I_{\gamma }^{}$ is the average integrated intensity of the XRD peaks of the austenite (200), (220) and (311) crystal planes; and $I_{\alpha }^{}$ is the average integrated intensity of the XRD peaks of the martensite (200) and (211) crystal planes.

Equation (2) was used to calculate the carbon content of the retained austenite [19,20]:

(2) $$C_{\gamma }^{}=\frac{a_{\gamma }^{}-3.556}{0.0453}$$

where $C_{\gamma }^{}$ is the carbon content of the retained austenite and $a_{\gamma }^{}$ is the lattice constant of the face-centered cubic (FCC) structure. The average value of the austenite (200), (220) and (311) lattice constants was used to calculate $a_{\gamma }^{}$ using Equation (3):

(3) $$a_{\gamma }=d\sqrt{h^{2}+k^{2}+l^{2}}$$

where *h*, *k*, *l* is a diffraction peak crystal plane index and *d* is the interplanar spacing, which can be calculated from the Bragg diffraction equation given below:

(4) 2dsinθ=λ

where *θ* is the diffraction angle and the wavelength of Cu irradiation λ is taken to be 1.54056 Å.

After low-temperature tempering, the strongest martensite peak shifted to higher angles. Equation (4) shows that the crystal plane spacing *d* decreases as the diffraction peak angle *θ* increases. Equation (3) shows that the lattice constant *a* decreases as the interplanar spacing *d* decreases. The decrease in the lattice constant reflected a reduction in the solid-solution carbon content of martensite.

The hardness is positively correlated with the carbon content in the martensite matrix. The higher the carbon content, the more severe the martensite lattice distortion is. Less distortion of the martensite lattice reduced the martensite contribution to the hardness, resulting in an overall decrease in the hardness of the carburized layer after the low-temperature tempering. The decrease in the solid-solution carbon content of the martensite in the matrix reflected the diffusion of carbon atoms in martensite during the low-temperature tempering, which may have enabled the formation of fine carbides, which precipitated or partitioned into the retained austenite [21].

**Figure 10:**
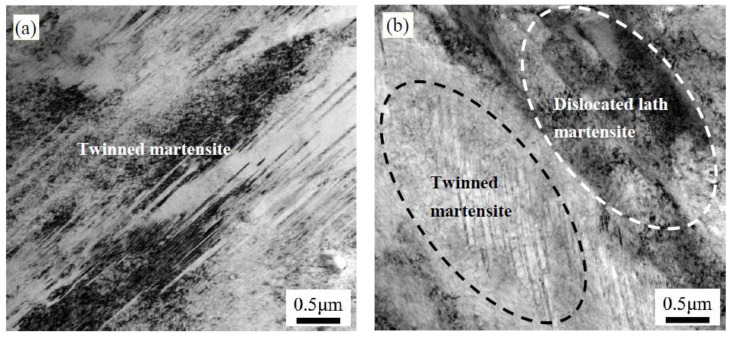
TEM images of the morphology of the martensite in the carburized layer: (**a**) after quenching; and (**b**) after the low-temperature tempering.

Figure 10 shows the TEM images of the morphology of the surface microstructure before and after the low-temperature tempering. The high-carbon content of the carburized layer resulted in the formation of high-carbon twin-type martensite in the carburized layer after quenching. After low-temperature tempering, the solid-solution carbon content in the martensite decreased, resulting in less twin-type martensite and a higher proportion of low-carbon lath martensite, which was consistent with the martensite XRD peak shifting to a higher angle. No significant carbide precipitates were found in the low-temperature tempered carburized layer, indicating that the carbon atoms in the large quantity of martensite were diffused into the retained austenite in solid-solution form [22,23].

Equations (1) and (2) were used to calculate the diffraction peak intensities. After carburizing and quenching, the retained austenite content was 24.91% and the carbon content in the retained austenite was 0.66%. After low-temperature tempering, the retained austenite content was reduced to 17.66% and the carbon content of the retained austenite increased to 0.74%. The volume expansion that occurs upon the transformation of austenite to martensite subjects the untransformed retained austenite to compressive stress, thereby improving the mechanical stability of the retained austenite. Low-temperature tempering reduced the extent of martensite lattice distortion, thereby lowering the compressive stress on the retained austenite. The corresponding reduction in the mechanical stability of the retained austenite resulted in some of the retained austenite transforming into martensite [18,21]. At the same time, some of the carbon atoms in the martensite diffused into the retained austenite, thereby raising the carbon content and enhancing the thermal stability of the retained austenite. As a result, some high-carbon retained austenite remained stable after low-temperature tempering.

### 4.2. Driving Force for Phase Transformation from Retained Austenite into Martensite

The thermal stability of the austenite in the carburized layer mainly depends on the carbon content and the temperature. The driving force for the phase transformation of austenite into martensite was calculated using the iron–carbon phase transformation KRC model proposed by Kaufman et al. [24,25]. In this section, the thermal stability of the retained austenite with different carbon contents is discussed from a thermodynamic perspective.

The driving force $\Delta G_{}^{\gamma \to \alpha }$ for the phase transformation of austenite to martensite (i.e., a supersaturated ferrite) with the same composition can be described as follows:

(5) $$\Delta G_{}^{\gamma \to \alpha }=(1-x_{\gamma }^{})\Delta G_{Fe}^{\gamma \to \alpha }+RT[x_{\gamma }^{}\ln\frac{a_{C}^{\alpha }}{a_{C}^{\gamma }}+(1-x_{\gamma }^{})\ln\frac{a_{Fe}^{\alpha }}{a_{Fe}^{\gamma }}]$$

where $\Delta G_{Fe}^{\gamma \to \alpha }$ represents the change in the partial molar free energy of pure iron under a γ→α phase transformation and is a function of temperature (the values presented in Mogutnov et al. [26,27] were used for calculation purposes); $a_{C}^{\gamma }$ is the activity of carbon in austenite; $a_{C}^{\alpha }$ is the activity of carbon in martensite; $a_{Fe}^{\gamma }$ is the activity of iron in austenite; and $a_{Fe}^{\alpha }$ is the activity of iron in martensite. The activities of carbon and iron atoms in austenite and ferrite can be described as follows:

(6) $$\ln a_{C}^{\gamma }=\ln\frac{x_{\gamma }}{1-z_{\gamma }x_{\gamma }}+\frac{\Delta {\bar{H}}_{\gamma }-\Delta {\bar{S}}_{\gamma }^{xs}T}{RT},$$

where $z_{\gamma }^{}$ is the interstitial coordination number, $z_{\gamma }^{}=14-12\exp(-w_{\gamma }/RT)$; $w_{\gamma }$ is the interaction energy of a pair of adjacent carbon atoms in austenite; $x_{\gamma }^{}$ is the mole fraction of carbon in austenite; $\Delta {\bar{H}}_{\gamma }$ and $\Delta {\bar{S}}_{\gamma }^{xs}$ are the changes in the partial molar enthalpy and the partial molar non-configurational entropy of carbon in austenite, respectively; R is the ideal gas constant (8.31 J/(mol·K)); and T is the absolute temperature. Shiflet et al. [28] performed numerous experiments to obtain $w_{\gamma }$ = 8054 J/mol, $\Delta {\bar{H}}_{\gamma }$= 38573 J/mol, and $\Delta {\bar{S}}_{\gamma }^{xs}$= 13.48 J/(mol·K):

(7) $$\ln a_{C}^{\alpha }=\ln\frac{x_{\alpha }}{3-z_{\alpha }x_{\alpha }}+\frac{\Delta {\bar{H}}_{\alpha }-\Delta {\bar{S}}_{\alpha }^{xs}T}{RT}$$

In the equation above, the coordination number of an interstitial site $z_{\alpha }$ is $z_{\alpha }^{}=12-8\exp(-w_{\alpha }/RT)$; $x_{\alpha }$ is the mole fraction of carbon in ferrite; and $w_{\alpha }$, $\Delta {\bar{H}}_{\alpha }$, and $\Delta {\bar{S}}_{\alpha }^{xs}$ are the interaction energy, the change in the partial molar enthalpy and the change in the partial molar non-configurational entropy of carbon in ferrite, respectively. Shiflet et al. [28] reported the following values: $w_{\alpha }$ = 8373 J/mol, $\Delta {\bar{H}}_{\alpha }$ = 112206 J/mol, and $\Delta {\bar{S}}_{\alpha }^{xs}$ = 51.46 J/(mol·K).

The chemical potentials of iron and carbon in austenite satisfy the Gibbs–Duhem equation ($x_{1}d\ln a_{1}+x_{2}d\ln a_{2}=0$). Thus, $a_{Fe}^{\gamma }$ can be obtained by integrating Equation (6):

(8) $$\ln a_{Fe}^{\gamma }=-\int_{0}^{x_{\gamma }}\frac{x_{\gamma }}{1-x_{\gamma }}d(\ln a_{C}^{\gamma })=\frac{1}{z_{\gamma }-1}\ln(\frac{1-z_{\gamma }x_{\gamma }}{1-x_{\gamma }})$$

Similarly, $a_{Fe}^{\alpha }$ can be obtained by the definite integration of the Gibbs–Duhem equation:

(9) $$\ln a_{Fe}^{\alpha }=-\int_{0}^{x_{\alpha }}\frac{x_{\alpha }}{1-x_{\alpha }}d(\ln a_{C}^{\alpha })=\frac{3}{z_{\alpha }-3}\ln[\frac{3-z_{\alpha }x_{\alpha }}{3(1-x_{\alpha })}].$$

The activities determined by Equations (6)–(9) can be substituted into Equation (5) to obtain the driving force for the decomposition of austenite to martensite within the KRC model [24,25]:

(10) $$\begin{array}{l} \Delta G_{}^{\gamma \to \alpha }=\frac{RT}{\left(z_{\alpha }-3)(z_{\gamma }-1\right)}[(z_{\gamma }-1)(3-z_{\alpha }x_{\gamma }^{})\ln(3-z_{\alpha }x_{\gamma }^{})\\ -(z_{\alpha }-3)(1-z_{\gamma }x_{\gamma }^{})\ln(1-z_{\gamma }x_{\gamma }^{})+(z_{\alpha }-3z_{\gamma })(1-x_{\gamma }^{})\ln(1-x_{\gamma }^{})\\ -3(z_{\gamma }-1)(1-x_{\gamma }^{})\ln3]+(1-x_{\gamma }^{})\Delta G_{Fe}^{\gamma \to \alpha }+x_{\gamma }^{}[\Delta {\bar{H}}_{\alpha }-\Delta {\bar{H}}_{\gamma }-(\Delta {\bar{S}}_{\alpha }^{xs}-\Delta {\bar{S}}_{\gamma }^{xs})T] \end{array}$$

**Figure 11:**
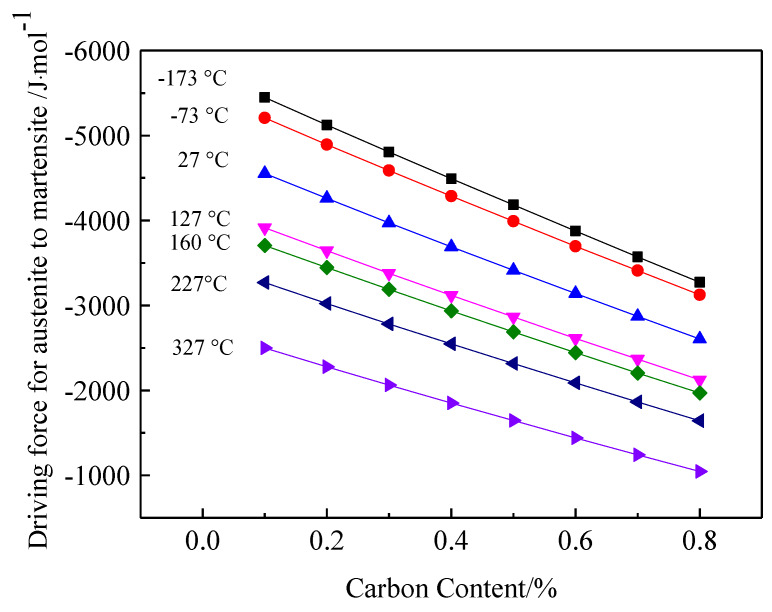
Driving force for the phase transformation of austenite into martensite.

Figure 11 shows the driving force for the phase transformation of austenite into martensite as a function of temperature and the carbon content. For a fixed carbon content, the driving force gradually increased with a decreasing temperature. At a fixed temperature, the driving force gradually decreased with an increasing carbon content. In brief, low-carbon and low-temperature conditions favored the transformation of retained austenite into martensite.

The thermodynamic calculation results showed that the driving force for the phase transformation into martensite was higher for austenite with a low-carbon content than for austenite with a high-carbon content. As carbon is an austenite-forming element, a high content of solid-solution carbon leads to a more stable austenite and a low driving force for the transformation into martensite. Therefore, after low-temperature tempering, some of the low-carbon retained austenite is likely to transform into martensite, whereas high-carbon retained austenite is more thermally stable than low-carbon retained austenite and is retained at room temperature.

Compared with that of low-carbon retained austenite, the calculated driving force for the phase transformation of high-carbon retained austenite only changed slightly with temperature, that is, the high-carbon retained austenite was less affected by temperature. As the temperature decreases, the increase in the carbon content of the carburized layer microstructure inhibits the transformation of the retained austenite into martensite.

Ishida [29] developed an empirical equation for determining the starting temperature Ms of the austenite into martensite transformation, as shown in Equation (11):

Ms (°C, wt%) = 545-330C + 2Al + 7Co-14Cr-13Cu-23Mn-5Mo-4Nb-13Ni-7Si + 13Ti + 4V + 0W(11)

Using Equation (11), the Ms of the 18Cr2Ni4WA steel core microstructure with 0.2% carbon was 389.7 °C; and the Ms of the outermost surface of the 18Cr2Ni4WA steel carburized layer with 0.67% carbon was approximately 234.6 °C, corresponding to a difference of 155.1 °C. The solid-solution carbon content in the matrix increases with the carbon content in the layer. Thus, the Ms transformation temperature is correspondingly lower, and the retained austenite has a strong thermal stability and is not likely to transform into martensite.

### 4.3. Effect of Cryogenic Treatments on the Microstructure and Case Hardness

The mechanical and thermal stability of the retained austenite was further investigated by introducing a −73 °C cryogenic treatment between quenching and low-temperature tempering, as shown in Figure 2b.

**Figure 12:**
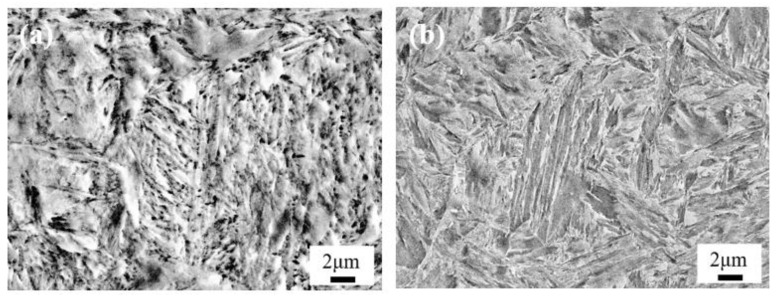
SEM images of the morphology of the carburized layer at a 0.3 mm depth for process 2: (**a**) after the quenching + cryogenic treatment; and (**b**) after the quenching + cryogenic treatment + tempering.

Figure 12 shows the SEM images of the structural morphology of the carburized layer at a 0.3 mm depth after the cryogenic treatment. The morphology of the carburized layer after the cryogenic treatment did not obviously change from the quenched microstructure shown in Figure 8a, and the block-shaped and lath-shaped austenite structures were distributed in the martensite matrix. After low-temperature tempering, the carbon content of the retained austenite decreased and needle-like and lath-like martensite structures were clearly observable. There was a negligible quantity of fine carbides in the microstructure.

**Figure 13:**
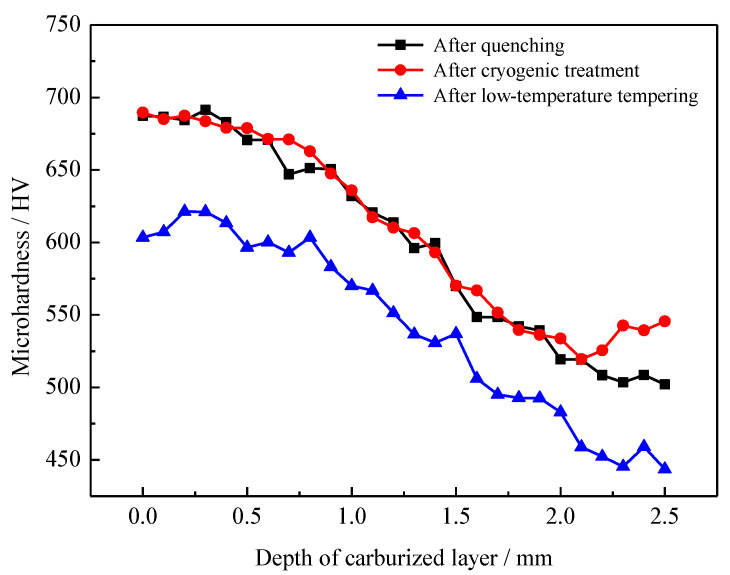
Hardness gradient of the carburized layer for process 2.

Figure 13 shows the hardness gradient of the carburized layer under the cryogenic treatment. After cryogenic treatment at −73 °C, the maximum hardness value of the surface of the carburized layer was 689 HV and the hardness of the core was 545 HV. After low-temperature tempering, the surface hardness of the sample carburized layer was 603 HV, the maximum hardness was 621 HV at a 0.2 mm depth, and the core hardness was 443 HV. After cryogenic treatment, the hardness of the carburized layer did not change and the hardness of the carburized layer decreased considerably after low-temperature tempering.

**Figure 14:**
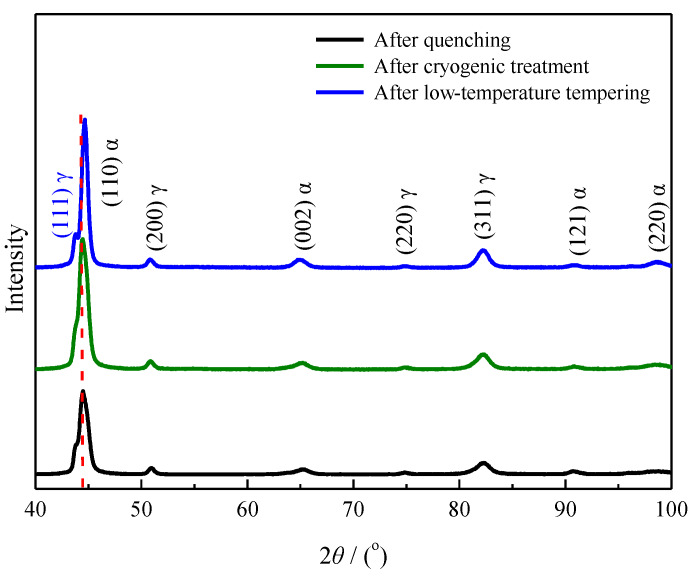
XRD patterns of the carburized layer at a 0.3 mm depth for process 2.

The XRD results obtained for the microstructure of the carburized layer at a 0.3 mm depth are shown in Figure 14. Compared to the XRD results after quenching, the cryogenic treatment moderately increased the peak intensity of the strongest peak corresponding to (110) martensite in the microstructure and did not considerably change the diffraction angle. Thus, the cryogenic treatment promoted the transformation of some retained austenite into martensite. After low-temperature tempering, the diffraction peaks of martensite (110) in the microstructure shifted to higher angles and the distinct diffraction peak of the austenite (111) crystal plane indicated that the carbon atoms in martensite diffused into the retained austenite.

**Figure 15:**
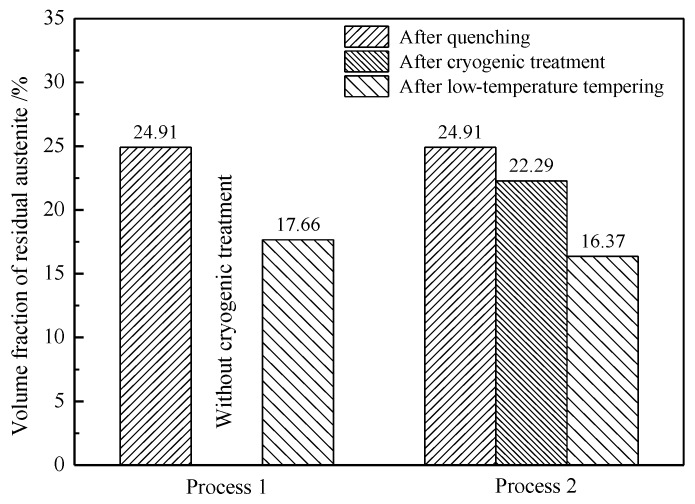
Volume fraction of the retained austenite in the carburized layer at a 0.3 mm depth.

**Figure 16:**
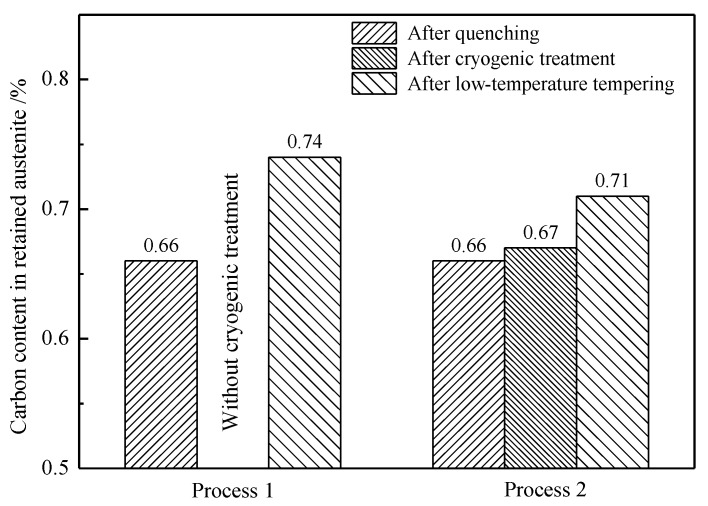
Carbon content of the retained austenite in the carburized layer at a 0.3 mm depth.

The volume fraction and carbon content of the retained austenite were calculated by substituting the XRD data into Equations (1) and (2). The results obtained for the different treatments are compared in Figure 15 and Figure 16.

The retained austenite content was reduced from 24.91% to 22.29% by the cryogenic treatment and further reduced to 16.37% by the low-temperature tempering. Thus, the cryogenic treatment had no apparent effect on the martensitic transformation and much more retained austenite was transformed by low-temperature tempering than by the cryogenic treatment. In addition to the temperature, other factors, such as stress, affected the transformation of the retained austenite. A large quantity of martensite was generated during the quenching process (after carburizing) and the resulting volume expansion generated a compressive stress on the untransformed retained austenite, suppressing further transformation. A higher driving force for the phase transformation is required to overcome this compressive stress to transform the retained austenite into martensite during the cryogenic treatment. Therefore, even if the temperature reaches the martensitic transformation temperature, the compressive stress inhibits the complete transformation of the retained austenite. The thermodynamic calculation results in Figure 11 show that the driving force for the phase transformation of the retained austenite with 0.6% carbon, increased from −3139 J/mol to −3697 J/mol when the temperature dropped from room temperature (27 °C) to −73 °C. The magnitude of the increase in the driving force was not large and there was no clear promotion of the martensitic transformation due to the temperature drop. As a relatively low quantity of the retained austenite transformed (as shown in Figure 15), the volume fraction of martensite did not change substantially, and the hardness did not significantly increase. The hardness of the carburized layer was not considerably improved after the cryogenic treatment, as shown in Figure 13.

The cryogenic treatment did not have a dramatic effect on the carbon content in the retained austenite, which increased from 0.66% to 0.67%. However, the low-temperature tempering significantly increased the carbon content in the retained austenite to 0.71%, indicating a considerable diffusion of carbon from the martensite into the retained austenite during tempering. The content of solid-solution carbon in martensite was reduced, the volume distortion was lessened and the martensite contribution to the hardness was significantly reduced. At the same time, the compressive stress on the retained austenite was reduced and the mechanical stability of the retained austenite decreased. Thus, as discussed in Section 4.2, some of the retained austenite with a low-carbon content transformed into martensite during the cooling period after tempering, since the high-carbon retained austenite was more thermally stable than the low-carbon retained austenite. Thus, the retained austenite with a high-carbon content was measured in the microstructure after tempering, as shown in Figure 16.

Therefore, the mechanical stability—not the thermal stability—of the retained austenite in the carburized layer dominated and the low-temperature tempering effectively promoted the transformation of the retained austenite. The changes in the martensite matrix hardness had a far greater effect than the transformation of the retained austenite into martensite on the microhardness of the carburized layer.

## 5. Conclusions

1. After carburizing and quenching, the carbon content of the carburized layer decreased gradually and evenly as the depth of the carburized layer increased. The matrix microstructure changed from high-carbon acicular martensite in the surface layer into low-carbon lath martensite in the core and the microhardness gradually decreased.
2. Calculating the driving force for the phase transformation of austenite showed that low-carbon and low-temperature conditions favored the transformation of retained austenite to martensite. Therefore, some low-carbon retained austenite was more likely to undergo martensitic transformation after low-temperature tempering.
3. During low-temperature tempering, the solid-solution carbon content of martensite decreased, the compressive stress on the retained austenite was reduced and the mechanical stability of the retained austenite decreased. Low-temperature tempering, rather than the cryogenic treatment, effectively promoted the transformation of the retained austenite.

## Figures and Tables

**Table 1:** Elemental content (mass fraction) of the 18Cr2Ni4WA steel.

Element	C	Si	Mn	Cr	Ni	W
Content	0.2	0.33	0.5	1.4	4.3	1.0
